# Volatile Compounds in Musk and Their Anti-Stroke Mechanisms

**DOI:** 10.3390/metabo15030181

**Published:** 2025-03-07

**Authors:** Chengli Zheng, Xin Shi, Qinling Yang, Zhongkun Cai, Xiao Wang, Liuqing Yang, Xue Bai, Xiuxiang Meng, Diyan Li, Hang Jie

**Affiliations:** 1 Sichuan Institute of Musk Deer Breeding, Sichuan Institute for Drug Control, Chengdu 611845, China; zhengcl@scidc.org.cn (C.Z.); shix@scidc.org.cn (X.S.); yanglq@scidc.org.cn (L.Y.); 2 Jinfo Mountain Forestry Ecosystem of Chongqing Observation and Research Station, Chongqing Institute of Medicinal Plant Cultivation, Chongqing 408435, China; 3 School of Pharmacy, Chengdu University, Chengdu 610106, China; caizhongkun@cdu.edu.cn (Z.C.);; 4 Shimadzu Enterprise Management (China) Co., Ltd., Chengdu 610023, China; 5 School of Ecology and Environment, Renmin University of China, Beijing 100872, China; 6 School of Resources and Environment, Aba Teachers College, Wenchuan 623002, China

**Keywords:** *Moschus berezovskii*, musk, volatile compounds, stroke, GC-MS

## Abstract

**Background/Objectives:** Musk is a widely used traditional Chinese medicine derived from musk deer that has the pharmacological effects of “activating blood dredging collateral” and “consciousness-restoring resuscitation”. Its volatile compounds (VCs) play a key role in these effects, especially in the treatment of stroke. However, there have been no comprehensive studies on the differences in the VCs of these different musks. This study investigated the differences in the VCs of different musks and the potential targets and mechanisms of action for stroke. **Methods:** Different musks were studied via GC–MS, and the potential targets and mechanisms of VCs associated with stroke were investigated using network pharmacology. **Results:** A total of 99 VCs were detected in 79 musk samples. The most important VCs of different colours and forms were muscone, phenol, acetic acid, and isovaleric acid. Further study revealed that the change in organic acids and ketones was the cause of the significant difference between white musk and other types of musk. In addition, network pharmacological analyses identified 180 potential targets of the major volatile compounds of musk associated with stroke, and five key targets (*SRC*, *EGFR*, *ESR1*, *PTGS2*, and *DRD2*). Enrichment analysis showed that these key targets play an important role in neural related pathways. The molecular docking results confirmed that the key targets can effectively bind with the main VCs (muscone and phenol). **Conclusions:** These findings provide valuable insights into the distinct volatile compositions of various types of musk and underscore the significant potential of volatile compounds (VCs) in stroke treatment.

## 1. Introduction

The forest musk deer (*Moschus berezovskii*) is an Asia-specific species of the Cervidae family renowned for the medicinal value of the musk secreted by the abdominal glands of males. Musk is a traditional Chinese medicinal ingredient known for its anti-inflammatory, analgesic, antiapoptotic, anticancer, and stress-relieving properties [1,2,3,4]. The peptides in musk (<10 kDa) were previously shown to have anti-inflammatory effects on a variety of inflammatory models [5]. Compound musk injection has been proven to have a positive restorative effect on hypoxic–ischaemic brain damage in newborns [6]. Muscone is considered to be the main active component of musk. Li et al. reported that high-dose muscone can reduce the atherosclerotic area and the formation of plaque collagen tissue in mice, and the mechanism of its effect may be related to antioxidant and anti-inflammatory mechanisms [7]. Another study confirmed that muscone repairs neurological damage caused by ischaemic brain injury by regulating the molecular mechanisms of neuronal synaptic connections [8]. The composition of musk is complex, and many of its active substances have not been effectively studied.

At present, the main technologies for the detection of musk components are gas chromatography–mass spectrometry (GC–MS) and liquid chromatography–mass spectrometry (LC–MS). Composition analysis has revealed that musk is composed of macrocyclic ketone compounds, amino acids, fatty acids, steroids, inorganic salts, and urea [9,10,11]. Musk is described in the Chinese Pharmacopoeia (2020 edition) as being used to treat “coma caused by critical illness” and “awakening the senses and reviving the spirit”. It is possible that certain volatile substances in musk penetrate the blood–brain barrier [8,12,13]. Through GC–MS analysis, Zhang et al. reported that musk contains volatile compounds such as alcohols, esters, ketones, and fatty acids [14]. Previous studies have used high-performance liquid chromatography to determine the main components of musk, including phenols, waxes, and other volatile compounds [15]. When collecting musk during production, we detected significant differences between individuals in terms of colour (black, white, brown, grey, and yellow) and morphology (powder, block, mud, strip, and paste). However, no relevant studies have compared the volatile compositions of these compounds.

Stroke, also known as cerebrovascular accident (CVA), is a critical medical condition characterised by the sudden disruption of blood flow to a part of the brain, leading to the rapid loss of neurological function [16]. CVA is a leading cause of long-term disability and mortality worldwide, affecting millions of people annually [17]. Research has shown that compound musk injection can treat ischaemic stroke (IS) by regulating pathways and targets related to the inflammatory response and apoptosis in a multicomponent manner [18]. Another study utilised ischaemia–reperfusion rat models to demonstrate that muscone, a volatile compound of musk, substantially decreased the incidence of cerebral infarction and mitigated tissue damage. Additionally, in vitro experiments confirmed that muscone significantly promoted the expression of neurotrophic and angiogenic factors [19]. In summary, current research primarily investigates the pharmacological effects of musk compound injections or single components, such as muscone, on stroke. However, the VCs in musk vary significantly across different types. We propose that the therapeutic efficacy of musk in treating stroke is likely due to the combined actions of multiple volatile compounds.

Therefore, in this study, GC–MS was used to compare and analyse the volatile compounds of 79 different musk types. The objective of this study was to investigate the differences in volatile chemical components of musks with different colours and forms, as well as their potential targets and mechanisms of action in stroke, to provide a reference for the use of natural musk.

## 2. Materials and Methods

### 2.1. Animals

We collected 79 musk samples from 79 musk deer (♂, 2–13 years old) from the Dujiangyan and Malkang farms. The collection of musk samples requires the collaboration of three individuals. The first person secures the forest musk deer, immobilizing its limbs to expose the lower abdominal scent sac organ. The second person then uses a specially designed spoon to extract the musk from the scent sac, while the third person collects the musk in a sampling tray, weighing out 1 g of musk to be placed in a sampling tube. It is important to note that we fully respect animal welfare and adhere to relevant guidelines for experimental sample collection. The musk collection process from the forest musk deer does not cause harm to the animal. In the event of any injuries sustained during the capture process, immediate disinfection with iodine is performed. The feed consisted of succulent (pumpkin, lettuce, carrot, cabbage, and white radish), pellet (trace element additives and dry leaves) and dry (elm leaves, mulberry leaves, and apricot leaves) feed. All individuals had no less than 8 m^2^ of activity space and ate and drank freely. The samples were collected in September 2024 and stored at −80 °C until use for follow-up testing.

### 2.2. Analysis of Volatile Compounds

#### 2.2.1. Sample Pretreatment

The headspace bottle was placed in the oven at 120 °C and allowed to bake for 30 min. Fifty milligrams of the sample to be tested was accurately weighed and put into the headspace bottle. After the bottle was sealed, it was prepared for analysis.

#### 2.2.2. Equipment Preparation

Before the formal experiment, we first tuned the GC–MS instrument (a process of performance optimisation and calibration). Next, we conducted three consecutive parallel tests for musk quality control samples and two consecutive parallel tests for blank samples to determine the instrument’s stability. A Shimadzu GC/MS-TO8050 NX (Shimadzu, Shanghai, China) triple quadrupole temperament combined with the AOC-6000 multifunction automatic injection device (Shimadzu, Shanghai, China) SPME injection method was used. The chromatographic column used was an InertCap Pure-WAX (Shimadzu, Shanghai, China) (30 m × 0.25 mm × 0.25 μm). The column temperature programme was as follows: maintain the initial temperature at 50 °C for 5 min, increase to 250 °C at a rate of 10 °C/min, and then maintain at 250 °C for 10 min. Helium was loaded at a constant pressure of 83.5 kPa. The injection method was a shunt injection with a shunt ratio of 5:1. The sample was tested when the instrument was properly prepared.

#### 2.2.3. Qualitative and Quantitative Characterisation of Volatile Compounds

First, the C9~C30 normal alkane standard was collected via the TQ_MS _Wax AART method using the odour analysis method package to calculate the retention times of the volatile substances. Three correction internal standards (4-bromofluorobenzene, 1,2-dichlorobenzene d4, and acenaphthene d10) were determined via the TQ_MS_Wax_Correct_MRM method. Using the above data and the Off-Flavour odour analysis database, a qualitative and semiquantitative method for identifying musk volatile substances was automatically established. Finally, 79 musk samples were tested using the method, and the qualitative and semiquantitative files of the volatile compounds were output. Each sample was tested in parallel 3 times. To ensure the precision of the matching process, we selected compounds with a similarity score of 80 or higher (on a scale of 0–100) and non-zero peak areas for further analysis.

### 2.3. Screening and Target Identification of Metabolites with Pharmacological Potential

All of the musk detection components were input into the PubChem database (https://pubchem.ncbi.nlm.nih.gov/, accessed on 10 January 2025) to determine the SMILES numbers. The compounds with potential pharmacological effects were identified through a literature search. The SEA search server (https://sea.bkslab.org/, accessed on 10 January 2025) and the Swiss Target Prediction (http://www.swisstargetprediction.ch/, accessed on 10 January 2025) database were subsequently used for target identification. The targets of the two databases were merged to remove duplicate values. In addition, construction and visualisation of the active material–target network was carried out via Cytoscape v3.10.3.

### 2.4. Prediction of Stroke-Related Targets

Stroke-related targets were searched with the keyword “stroke” in two databases: OMIM (https://omim.org/, accessed on 12 January 2025) and GeneCards (https://www.genecards.org/, accessed on 12 January 2025). The search criterion was a relevance score ≥ 5 in the GeneCards database. Stroke-related targets from the two databases were finally merged, and duplicate values were removed.

### 2.5. PPI Network Between Musk Metabolite Targets and Stroke Targets

First, the crossover targets of the musk metabolite (muscone, phenol, and isovaleric acid) targets and stroke targets were obtained through the online website Hiplot (https://hiplot.com.cn/home/index.html, accessed on 15 January 2025) and visualised using a Venn diagram. The cross-targets were subsequently imported into the STRING database (http://string-db.org/, accessed on 15 January 2025) to construct the PPI network. The minimum interaction threshold was set to 0.4, and the other parameters were set to default values [20]. The adjustment and visualisation of the PPI network were performed in the STRING database and can be downloaded for use.

### 2.6. Molecular Docking

The SDF-formatted ligand files for the volatile compounds were obtained from the PubChem database (https://pubchem.ncbi.nlm.nih.gov/, accessed on 20 January 2025), and the three-dimensional structure models of the proteins EGFR (PDB ID: 1XKK), PTGS2 (PDBID: 3NT1), SRC (PDB ID: 8BQ3), DRD2 (PDB ID: 8IRS), and ESR1 (PDB ID: 6DFN) were obtained from the PDB database (https://www.rcsb.org/, accessed on 20 January 2025). The preprocessing of the protein and ligand preceded molecular docking. The protein’s three-dimensional structure was first imported into PyMOL v4.6.0, where water molecules, metal ions, and specific ligands were removed and the structure was saved. The molecular docking involved (1) opening the protein structure in AutoDockTools v1.5.6 to add hydrogen atoms and charges; (2) opening the three-dimensional structure of the ligand and selecting the option to enable bond rotation; (3) selecting the “Grid box” to encompass the protein model, ensuring energy scoring for all binding sites; and (4) using AutoDock Vina v1.1.2 to predict molecular docking, resulting in 9 conformations. The visualisation of protein–ligand docking was achieved with PyMOL by opening the ligand and conformation files and selecting the optimal conformation based on binding energy and polar contact sites.

### 2.7. Statistical Analysis

The correlation plot and significance analysis were generated via the R software (v.4.2.2) package “corrplot” (v.0.92) and package “ggpubr” (v0.4.0), respectively. Pearson’s correlation calculation was employed, and significance was denoted by *p*-values, with * indicating *p* < 0.05, ** indicating *p* < 0.01, and *** indicating *p* < 0.001. The average peak areas of 99 VCs in different types of musk were used as input data for the correlation analysis. The R package “ggplot2” (v3.4.2) was used for data visualisation. We used Student’s *t*-test to analyse the significance of changes in the contents of ketones and organic acids in different types of musk and presented the results as box plots. GO and KEGG enrichment were performed with the online site Metascape (https://metascape.org/, accessed on 21 January 2025) for analysis. The chosen parameters included a minimum overlap of ≥3, a *p*-value less than 0.01, and an enrichment score exceeding 1.5. The results were visualised with Hiplot (https://hiplot.com.cn/, accessed on 24 January 2025).

## 3. Results

### 3.1. Phenotypes of Musks with Different Colours and Shapes

**Figure 1:**
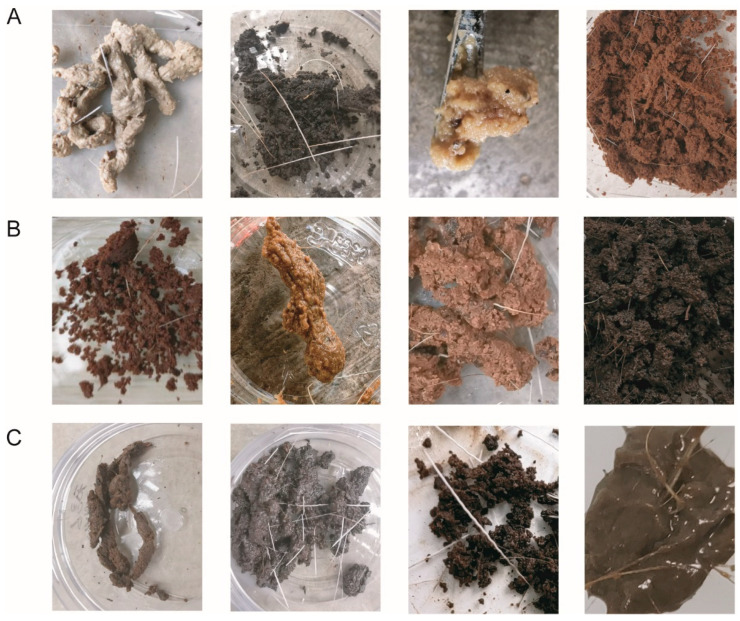
Phenotypes of musks of different colours and shapes. From left to right: (**A**) white, black, yellow, and brown; (**B**) transitional colours of red-brown, yellow-brown, light brown, and dark brown; (**C**) strips, ointments, powders, and pastes.

The musk samples we collected were classified into black, white, yellow, brown, and some transitional colours (yellow-brown, red-brown, light brown, and dark brown) (Figure 1A,B). According to their morphological classification, the musks were categorised as strips, ointments, powders, and pastes (Figure 1C).

### 3.2. Main Volatile Compounds of Musk

**Figure 2:**
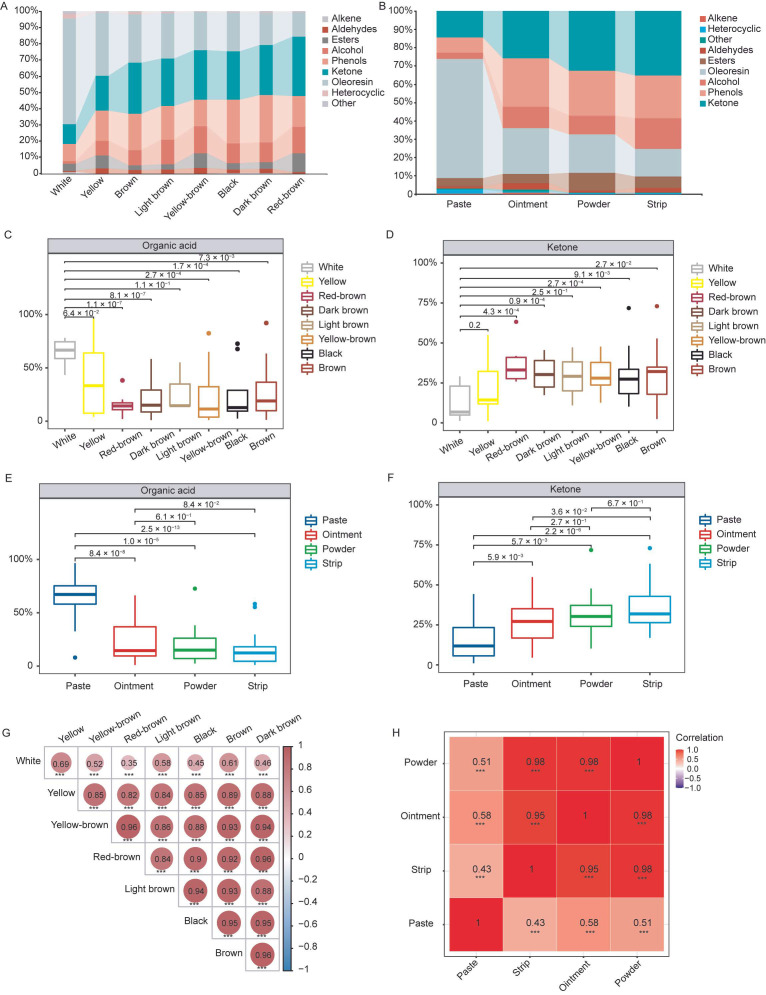
Main volatile compounds of natural musk. (**A**) Proportion of compounds in musks of different colours. (**B**) Proportion of musk compounds in different forms. Proportions of organic acids (**C**) and ketones in musks of different colours (**D**). Proportions of organic acids (**E**) and ketones (**F**) in musks with different morphologies. Correlation heatmap of musks of different colours (**G**) and morphologies (**H**) according to musk component average peak areas. Correlation calculation method = Pearson’s r, with *** indicating *p* < 0.001.

Prior to the introduction of the samples, we optimised and calibrated the GC–MS instrument, ensuring that it functioned within the established parameters (Supplementary Figure S1A). Subsequently, three parallel injections of the quality control samples were performed, revealing excellent overlap in retention times and peak areas, indicative of instrument stability (Supplementary Figure S1B). Furthermore, the analysis of blank samples showed no appreciable peaks, confirming the effective control of the material residue and the absence of cross-contamination between samples (Supplementary Figure S1B). A total of 99 volatile compounds of different groups of musk were successfully identified via GC–MS (Supplementary Tables S1-1 and S1-2). These compounds were divided into nine compound classifications, with the top three being 26 alcohols, 14 esters, and 14 ketones (Supplementary Figure S1C). According to the different proportions of the metabolite peak area, we found that organic acids accounted for the highest proportion of the total metabolites in white musk (65.28%), followed by other musks, and the lowest proportion in red-brown musk (15.18%) (Figure 2A). In contrast, the ketone content was the lowest in white musk (12.29%) and higher in other musks, with the highest observed for red-brown musk (36.60%) (Figure 2A). After analysing the musks as paste, ointment, powder, and strips according to their physical forms, we found that the proportion of organic acid in paste musk was the highest (65.09%) and showed a downward trend, reaching the lowest value in strips (15.07%) (Figure 2B). However, the proportion of ketones in paste musk was the lowest (14.64%), which showed an increasing trend and reached the highest value in strip musk (35.19%) (Figure 2B). Further analysis revealed that the proportion of organic acids in white musk was significantly greater than that in the other colours of musk, with the exceptions of yellow and light brown (Figure 2C). Additionally, the ketone content in white musk was notably lower than that in red-brown, dark brown, yellow-brown, brown, and black musk (Figure 2D). The organic acid content in paste musk was significantly higher than that in ointment, powder, and strip musk, with a gradual downward trend (Figure 2E). In contrast, its ketone content was markedly lower than in these forms, exhibiting a gradual upward trend (Figure 2F). The correlation heatmaps revealed that yellow musk, yellow-brown musk, red-brown musk, light brown musk, black musk, dark brown musk, and brown musk were highly correlated (*r* > 0.82), indicating that their compositions and contents are similar (Figure 2G). However, white musk had a low correlation with other-coloured musks (*r* < 0.69) and a large difference in composition (Figure 2G). When a correlation analysis was conducted on the basis of morphological classification, it was found that strip musk, ointment musk, and powder musk were highly correlated (*r* > 0.95), and their compositions and contents were similar, whereas paste musk and other forms of musk had large differences in composition and content (*r* < 0.58) (Figure 2H). Notably, the percentage of white musk in paste musk was significantly greater than that in the other colours (62.5%, 15/24) (Supplementary Figure S1D). These results indicate that the change in musk colour from light to dark may be accompanied by a decrease in organic acid content and an increase in ketones.

### 3.3. Signature Volatile Chemical Compounds of Musks of Different Colours and Forms

**Figure 3:**
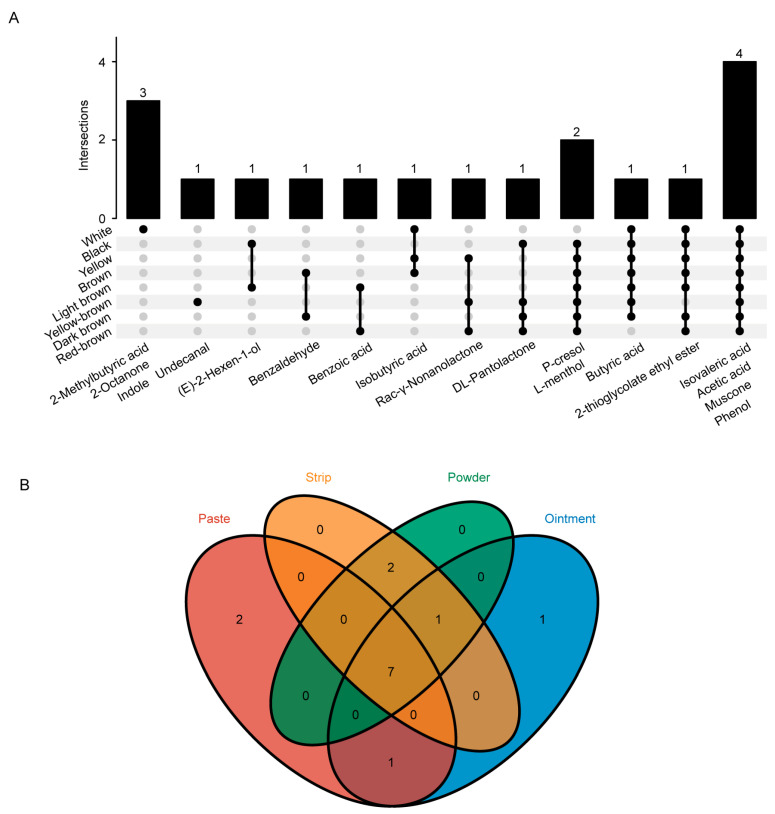
Signature chemical composition of musk in different colours and forms. (**A**) UpSet analysis of the volatile chemical constituents of the top 10 musk samples of different colours. Each column represents a set of given compounds. (**B**) Venn diagram of the top 10 volatile chemical constituents of different types of musk.

To understand the signature volatile compounds of musks of different colours and forms, we sorted them according to the proportion of the peak area. The results revealed that, of the top 10 volatile compounds in the musks of different colours, the most common were phenol, acetic acid, muscone, and isovaleric acid (Figure 3A). Among the top 10 volatile compounds of different musk forms, the seven most common were butyric acid, acetic acid, isovaleric acid, muscone, phenol, 2-thioglycolate ethyl ester, and p-cresol (Figure 3B). Muscone, phenol, acetic acid, and isovaleric acid were the most common volatile chemical compounds of musks of different colours and forms. The top 10 volatile chemical compounds accounted for 90.84% of the white musks, 86.88% of the yellow musks, 90.12% of the brown musks, 91.51% of the black musks, 87.52% of the light brown musks, and 91.17% of the dark brown musks. For the yellow-brown colour, the amount was 90.57%, and for the red-brown colour, it was 95.55%. The top 10 volatile chemical compounds accounted for 84.33% of the ointment musk, 87.82% of the paste musk, 91.59% of the strip musk, and 94.09% of the powder musk. These substances are signature volatile compounds that represent musks of different colours and forms, and are shown in Table 1 and Table 2.

### 3.4. Potential Mechanism of the Signature Volatile Compounds of Musk Against Stroke

#### 3.4.1. Target Prediction and Critical Target Screening

**Figure 4:**
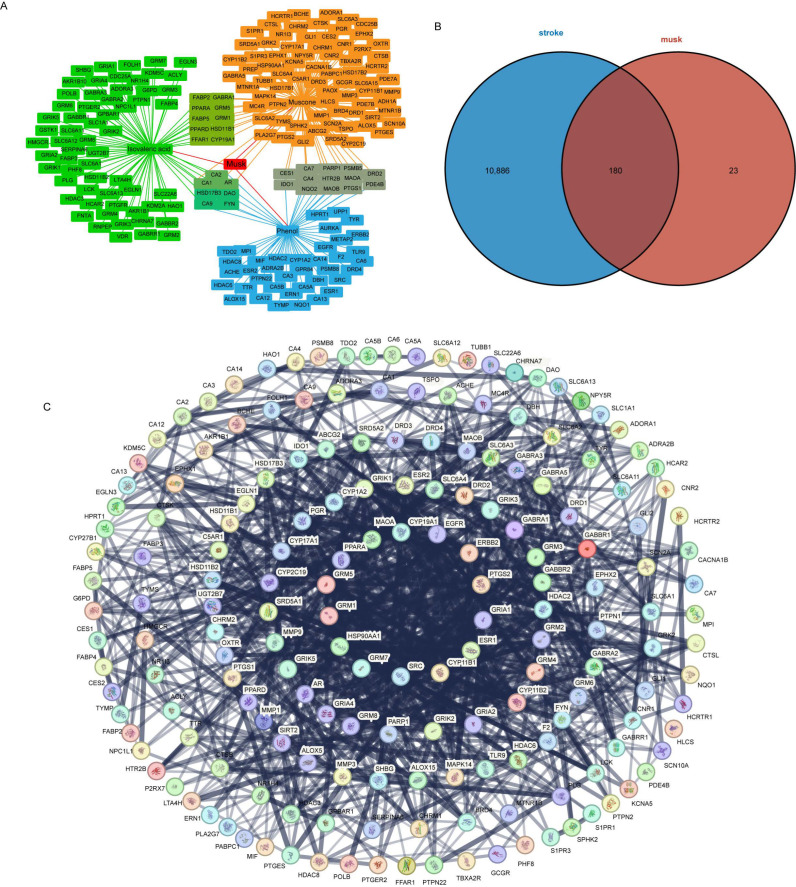
Musk–target–disease interaction network analysis. (**A**) Construction of the musk–volatile compound–target network. (**B**) Venn diagram of the predicted volatile compound targets and stroke-related targets. (**C**) PPI network of intersecting targets. Note: (**A**,**B**) Diagrams with larger dimensions and higher resolution are displayed in the Supplementary Materials (Figure S1E,F).

The volatile chemical compounds (acetic acid, isovaleric acid, phenol, and muscone) common to different types and forms of musk were selected for a network pharmacological analysis. Using the SEA and Swiss Target Prediction databases, a total of 236 targets of three volatile compounds were predicted (acetic acid did not predict the target). The musk–volatile compound–target network was visualised via Cytoscape v3.10.3 (Figure 4A). After the removal of duplicates, 203 specific targets were identified (Supplementary Table S2). In addition, 11,040 stroke-related targets were predicted via the GeneCards database, and 404 were predicted via the OMIM database. A total of 11,066 stroke-related specific targets were obtained after removing duplicates (Supplementary Table S3). A Venn diagram was used to identify the intersection target between the predicted musk target and the stroke target. The analysis identified 180 potential anti-stroke targets (Figure 4B). The PPI network for the 180 intersecting targets was constructed using the STRING database (Figure 4C, Supplementary Table S4). The PPI network comprised 180 nodes and 1192 edges. The average node degree of the PPI network was 13.2, and the interaction *p*-value was <1.0 × 10^−16^, which means that the PPI network has significant biological significance. The degree value represents the number of edges connected to a node, which indicates the strength and confidence of the interaction. Among the targets, *SRC*, *EGFR*, *ESR1*, *PTGS2*, and *DRD2* presented the highest degree values, with degrees of 59, 47, 44, 43, and 38, respectively, identifying them as potential key targets (Supplementary Table S4). 

#### 3.4.2. Functional Enrichment Analysis of Key Targets

**Figure 5:**
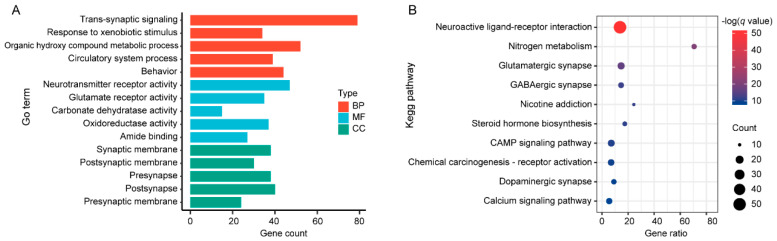
GO and KEGG enrichment analyses of key targets. (**A**) GO enrichment analysis of the top five enriched terms. (**B**) KEGG pathway analysis of the top 10 enriched terms.

To comprehensively understand the mechanisms through which musk volatile compounds act on stroke at the molecular level, GO and KEGG enrichment analyses were performed on the 180 intersecting targets. The results of the GO enrichment analysis revealed that the key targets were significantly enriched in 255 categories, including BP (169), MF (51), and CC (35) (Supplementary Table S5). These genes were enriched mainly in neurotransmitter receptor activity (GO:0030594), transsynaptic signalling (GO:0099537), glutamate receptor activity (GO:0008066), response to xenobiotic stimulus (GO:0009410), and the synaptic membrane (GO:0097060) and postsynaptic membrane (GO:0045211). The enrichment results for the top five genes in each category are shown in Figure 5A. Through KEGG enrichment analysis, 180 key targets were enriched in 63 signalling pathways (Supplementary Table S6). These genes were mainly enriched in the neuroactive ligand–receptor interaction (hsa04080), glutamatergic synapse (hsa04724), and GABAergic synapse (hsa04727) pathways (Figure 5B). The results of the KEGG pathway and GO enrichment analyses suggest that the key targets play important roles in signalling processes such as pre- and postsynaptic membrane signal reception, nerve transmembrane signal transmission, and the regulation of related reactive enzyme activity.

#### 3.4.3. Molecular Docking Verification

**Figure 6:**
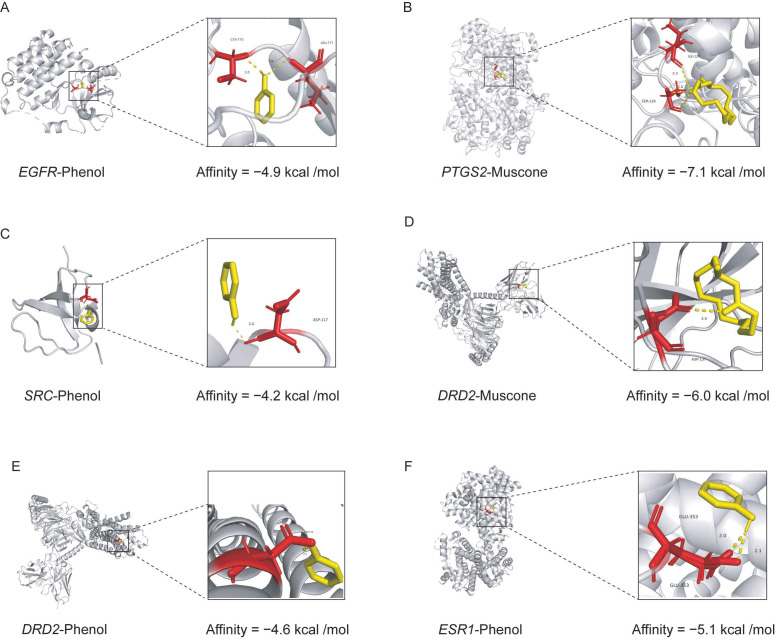
Molecular docking of the main volatile compounds with *EGFR*, *PTGS2*, *SRC*, *DRD2*, and *ESR1*. (**A**) Interaction between EGFR and phenol. (**B**) Interaction between PTGS2 and muscone. (**C**) Interaction between SRC and phenol. (**D**) Interaction between DRD2 and muscone. (**E**) Interaction between DRD2 and phenol. (**F**) Interaction between ESR1 and phenol.

Molecular docking methods were used to evaluate the binding affinity of volatile constituents to the top five key target genes (*EGFR*, *PTGS2*, *SRC*, *DRD2*, and *BCL2*). We constructed molecular docking complexes based on previously predicted VC targets: *EGFR*–Phenol, *PTGS2*–Muscone, *SRC*–Phenol, *DRD2*–Muscone, *DRD2*–Phenol, and *ESR1*–Phenol (Supplementary Tables S2 and S4). Depending on the structure of the binding protein, phenol has different binding sites and activities with *EGFR*, *SRC*, *DRD2*, and *ESR1*. Phenol has a binding site with *SRC* (ASP-117) and *DRD2* (ASP-114) with binding affinities of −4.2 kcal/mol and −4.6 kcal/mol, respectively (Figure 6C,E). Phenol also has two binding sites with *EGFR* (CYS-775 and LEU-777) and *ESR1* (GLU-353), with binding activities of −4.9 kcal/mol and −5.1 kcal/mol, respectively (Figure 6A,F). There are three binding sites of muscone to *PTGS2*, which are linked to ILE-124 and SER-126, and the binding affinity is −7.1 kcal/mol (Figure 6B). In addition, muscone and *DRD2* have a binding site at ASP-137 with a binding affinity of −6.0 kcal/mol (Figure 6D). These results indicate that the volatile chemical compounds of musk can exert anti-stroke effects by binding to stroke targets.

## 4. Discussion

As a traditional Chinese medicine, natural musk has been widely used for its anti-inflammatory, bactericidal, and antitumour effects and for the treatment of cardiovascular and nervous system diseases [19,21,22]. In particular, in the fields of “activating blood dredging collateral” and “consciousness-restoring resuscitation”, the effect is remarkable. For example, the “Angong Niuhuang pill”, which is made of musk as the main material, has a marked effect on the treatment of stroke [23]. These findings suggest an important role for the volatile compounds of musk in the treatment of stroke. However, owing to the variety of colours and forms of natural musk, no studies have fully analysed the differences in volatile compounds. Therefore, this study aimed to comprehensively analyse the differences in volatile compounds of musks of different colours and forms through GC–MS technology and to explore the potential targets and mechanisms of action of these volatile compounds in the treatment of stroke through network pharmacology.

### 4.1. Differences in Volatile Compounds Among Different Musk Phenotypes

Using the GC–MS technique, we analysed the volatile compounds of musks of different colours and forms, and a total of 99 volatile substances were obtained from 79 musk samples. According to the different functional groups, there were nine types of substances, among which alcohols were the most common with 26 types, followed by 14 types of esters and ketones (Supplementary Figure S1C). This is similar to the composition of musk metabolites reported in other studies [11,15]. By analysing the differences in the volatile compounds of different coloured musks, we found that red-brown musks, light brown musks, black musks, dark brown musks, brown musks, and yellow-brown musks were highly correlated with each other, whereas white musks were poorly correlated with each other. Further studies revealed that the contents of ketones and organic acids in white musks were significantly different from those in other musks (Figure 2). Ketones are the main pharmacologically active substances of musk, especially the content of muscone, which often affects the quality of musk [24,25,26]. Among the VCs studied for their efficacy against stroke, muscone has received the most attention and has been shown to be highly effective in stroke treatment [19,27,28,29,30]. Considering muscone content as a key quality indicator for musk’s effectiveness in treating stroke, our findings from Figure 2 suggest that white musk is the least effective, while red-brown musk is the most effective, corresponding to their respective low and high muscone concentrations. We believe that white musk is likely to be an unqualified abnormal musk; this view is consistent with that of Zhang et al. [14]. In the actual collection of musk, the odour emitted by white musk is unpleasant and is the result of the volatile compounds in the musk. The results of our identification indicate that the odour of white musk is caused mainly by the organic acid content, which is significantly greater than that of other coloured musks. In addition, the changing trend in the volatile component content indicates that the colour of musk gradually intensifies from white; we found that the initial musk obtained from forest musk deer is an almost milky-white liquid, and after a period of maturation in the sachet, the musk exhibits a different colour [31]. However, the colour formation mechanism of musk has not been fully elucidated. Our previous studies revealed that microorganisms in the musk sachet also participate in the maturation of musk, so it is speculated that this may be caused by the joint action of substances secreted by the musk gland itself and microorganisms in the musk sachet [10,31].

After the classification and analysis of the musks according to morphology, we found that different types of musk have more similar volatile compounds (Figure 2H and Figure 3B), except for paste musk. When we further analysed the composition of paste musk, we found that white musk was present in almost all paste musk, except for two ointment musks. This led us to associate white musk with paste musk (Supplementary Figure S1D and Table S1-2). This finding, combined with the above description of the initial musk, leads us to speculate that white paste musk has the worst-quality fragrance and that the process of maturation is lacking, the mechanism of which needs to be further studied.

### 4.2. Potential Targets and Mechanisms of Musk Volatile Compounds and Stroke

Musk is known for its marked effects on stroke [28,32,33]. In the second part of this study, to explore the uniformity of different musk types in treating stroke, we identified four common substances shared among the top 10 compounds of musks of various types (colour, form): muscone, phenol, isovaleric acid, and acetic acid. Muscone is considered the main active substance of musk and has been proven to have a protective effect on nerve damage after stroke [29,30]. Phenol, an intriguing compound that we identified, is consistently found among the top 10 compounds across various musk types. It is well known that phenol has strong corrosiveness and toxicity. Skin contact with phenol can cause protein denaturation and scarring and, after being absorbed by the body, it may cause damage to heart, liver, and kidney functions [34]. Despite its risks, the phenol scaffold, as a structural unit, exhibits a variety of beneficial pharmacological effects, including antioxidant, antiviral, antitumor, antibacterial, anti-carcinogenic, anti-mutagenic, and anti-inflammatory properties [35]. Previous studies have utilised phenol for the treatment of stroke sequelae, a method referred to as phenol neurolysis. Research by Mas and colleagues showed that injecting phenol after a stroke can alleviate early muscle spasms without causing additional side effects [36]. Li et al. [37] studied the use of phenol neurolysis under ultrasound and electrical stimulation guidance. The results showed that it can successfully alleviate focal spasms caused by conditions such as stroke. Fifty-seven patients received a total of 139 neurolysis procedures, with only three cases of persistent pain reported. However, no touch-evoked pain was reported during the follow-up period. These findings demonstrate that phenol has the potential for pharmacological effects, whether in its derivatives or in its native form. Nevertheless, given the severe toxicity of phenol, it is crucial to assess the phenol content when musk is employed in medicinal preparations. Our findings indicated that the maximum concentration of phenol in the different musk samples was no more than 10%. Furthermore, it is essential to be vigilant regarding the presence of other potentially toxic compounds within musk samples. Acetic acid and isovaleric acid are short-chain fatty acids. Fatty acids are essential components of musk. Our recent study provides evidence for positive selection and rapid evolution in the lipid metabolism pathway-related genes in forest musk deer, which explains the driving mechanism behind the abundance of fatty acids in musk [38]. Previous studies have found that short-chain fatty acids derived from intestinal microorganisms can improve neurological deficits and inflammation in mice and rats after stroke [39,40]. Similarly, our previous research found that microorganisms are involved in the maturation process of musk and, as a result, may produce short-chain fatty acid by-products [10]. Therefore, we speculate that the short-chain fatty acids in musk might also have a therapeutic effect on stroke similar to that of short-chain fatty acids derived from intestinal microorganisms. A total of 180 crossover targets were obtained via intersection analysis between the common VCs of different-coloured musks and stroke targets. GO enrichment analysis revealed that key targets were mainly enriched in biological functions related to the nervous system, such as neurotransmitter receptor activity, transsynaptic signalling, the synaptic membrane, and the postsynaptic membrane. KEGG enrichment analysis revealed that key targets were mainly enriched in neuroactive ligand–receptor interactions, glutamatergic synapses, GABAergic synapses, and other signalling pathways. Strokes are classified as ischaemic [41] or haemorrhagic [42], both of which cause severe nerve damage to the brain. Therefore, the anti-stroke effect of the volatile compounds in musk is mediated by a variety of biological pathways related to the nervous system.

PPI network analysis revealed the top five potential key target proteins: *SRC*, *EGFR*, *ESR1*, *PTGS2*, and *DRD2*. These genes may target the volatile compounds of musk to play an anti-stroke role. Studies have shown that *SRC* regulates smooth muscle contraction after stroke, which leads to increased cerebrovascular tension and poststroke reperfusion injury [43]. Cognitive decline in patients with ischaemic stroke has been found to be associated with reduced *EGFR* expression, which is considered to be an effective indicator of cognitive impairment in stroke patients [44]. Studies have shown an association between *ESR1*, or oestrogen receptor 1, and stroke, with a significantly increased risk of stroke in subjects with the *ESR1* c.454-397CC genotype [45]. *PTGS2* has been shown to reduce neurological damage caused by ischaemic stroke via the NF-kappa B axis [46]. *DRD2*, a dopamine receptor gene, is expressed in the microglia and macrophages in the brains of stroke mice and may help regulate the neuroinflammation caused by stroke [47]. Therefore, *SRC*, *EGFR*, *ESR1*, *PTGS2*, and *DRD2* are closely related to the occurrence and development of stroke, and the volatile compounds of musk may treat nerve damage caused by stroke by binding to these key target proteins. We then used calculations to model the binding affinity and mechanisms of the volatile compounds of musk to key stroke-related targets. The results revealed that *PTGS2*–muscone (ILE-124 and SER-126), *DRD2*–muscone (ASP-137), and *ESR1*–phenol (GLU-353) were the best volatile substance–ligand complexes, with binding activities of less than −5 kcal/mol.

## 5. Conclusions

In this study, metabolomics analysis via gas chromatography–mass spectrometry (GC–MS) was employed to characterise the volatile chemical constituents of musks with varying colours and morphologies. It was determined that ketones and organic acids are pivotal factors influencing the distinct types of musk. Through network pharmacology and computational simulations, key targets associated with musk volatile compounds and stroke, including *SRC*, *EGFR*, *ESR1*, *PTGS2*, and *DRD2*, were identified. Molecular docking studies demonstrated that these key targets can bind effectively to the major volatile compounds of musk. These findings are anticipated to provide valuable insights into the utilisation of natural musk in anti-stroke therapies.

## Figures and Tables

**Table 1:** Presence of top 10 signature volatile compounds according to musk colour.

No.	Chemical Name	CAS	White	Yellow	Brown	Black	Light Brown	Dark Brown	Yellow-Brown	Red-Brown
1	Butyric acid	107-92-6	24.64%	9.05%	10.47%	8.11%	17.13%	3.26%	7.14%	0.55%
2	Isovaleric acid	503-74-2	15.26%	9.85%	11.26%	7.97%	2.08%	8.28%	8.37%	6.57%
3	Acetic acid	64-19-7	9.68%	16.71%	4.45%	5.74%	6.02%	7.09%	5.72%	7.23%
4	Phenol	108-95-2	9.49%	6.59%	6.77%	1.44%	2.10%	9.96%	6.67%	4.54%
5	Muscone	541-91-3	9.36%	19.00%	29.54%	28.14%	26.32%	28.82%	28.31%	35.56%
6	Isobutyric acid	79-31-2	6.58%	1.90%	1.72%	0.87%	1.15%	0.49%	1.17%	0.00%
7	2-Methylbutyric acid	116-53-0	5.80%	0.00%	0.46%	0.00%	0.00%	0.00%	0.00%	0.00%
8	2-thioglycolate ethyl ester	623-51-8	3.48%	3.18%	1.72%	2.63%	2.33%	3.14%	1.94%	3.85%
9	2-Octanone	111-13-7	2.41%	1.57%	1.21%	1.10%	1.54%	0.89%	0.90%	0.64%
10	Indole	120-72-9	2.14%	0.00%	0.85%	0.00%	0.00%	0.13%	0.00%	0.00%
11	P-cresol	106-44-5	1.04%	11.78%	15.83%	25.01%	18.49%	17.82%	9.11%	13.79%
12	L-menthol	2216-51-5	0.09%	5.29%	6.78%	8.81%	7.10%	10.58%	15.81%	15.69%
13	Rac-γ-Nonanolactone	104-61-0	0.01%	3.53%	0.61%	0.00%	0.00%	0.00%	4.56%	3.78%
14	(E)-2-Hexen-1-ol	928-95-0	0.04%	0.50%	0.66%	2.22%	4.26%	0.30%	0.28%	0.00%
15	Benzoic acid	65-85-0	0.21%	0.43%	0.27%	0.37%	1.69%	0.55%	0.66%	0.82%
16	Undecanal	112-44-7	0.00%	1.30%	0.00%	0.00%	0.00%	0.95%	2.57%	0.00%
17	DL-Pantolactone	79-50-5	0.00%	0.11%	0.54%	1.44%	1.10%	1.12%	2.31%	3.72%
18	Benzaldehyde	100-52-7	0.55%	0.89%	1.58%	1.36%	0.98%	1.10%	0.24%	0.36%

Note: The values represent the percentage of the composition of the substance in the sum of the peak areas of all components. The grey areas are the top 10 substances in each sample.

**Table 2:** Presence of top 10 signature volatile compounds according to musk form.

No.	Chemical Name	CAS	Ointment	Paste	Strip	Powder
1	Muscone	541-91-3	23.94%	11.56%	33.53%	31.43%
2	P-cresol	106-44-5	18.26%	2.07%	16.40%	17.48%
3	L-menthol	2216-51-5	7.68%	1.48%	15.09%	9.40%
4	Phenol	108-95-2	7.64%	6.08%	6.42%	6.38%
5	Isovaleric acid	503-74-2	7.44%	13.30%	8.21%	10.25%
6	Butyric acid	107-92-6	6.63%	26.20%	2.24%	3.33%
7	Acetic acid	64-19-7	6.45%	14.09%	3.65%	6.31%
8	2-thioglycolate ethyl ester	623-51-8	2.74%	3.68%	1.81%	3.47%
9	Isobutyric acid	79-31-2	1.88%	5.45%	0.15%	0.29%
10	(E)-2-Hexen-1-ol	928-95-0	1.67%	0.35%	0.53%	0.00%
11	2-Methylbutyric acid	116-53-0	1.03%	3.25%	0.17%	0.00%
12	2-Octanone	111-13-7	1.38%	2.14%	0.83%	0.73%
13	DL-Pantolactone	79-50-5	0.67%	0.06%	2.31%	2.22%
14	rac-γ-Nonanolactone	104-61-0	1.29%	0.20%	1.93%	3.82%

Note: The numbers in the columns represent the ratio of the peak area of this metabolite to the total peak area of all metabolites in the sample. The grey areas are the top 10 substances in each sample.

## Data Availability

The original contributions presented in this study are included in the article and Supplementary Materials. Further inquiries can be directed to the corresponding authors.

## Supplementary Materials

The following supporting information can be downloaded at: https://www.mdpi.com/article/10.3390/metabo15030181/s1, Figure S1A: The tuning results for the GC–MS instrument. Figure S1B: Measurement peak profiles of quality control samples and blank samples. Figure S1C: Different classifications of VCs. Figure S1D: Proportion of different-coloured musks in paste musk. Figure S1E: Construction of the musk–VC–target network. Figure S1F: PPI network of intersecting targets. Table S1-1: GC–MS detection data for the musk sample. Table S1-2: Musk sample corresponds to colour and shape. Table S2: Targets of VCs predicted using SEA and Swiss Target databases. Table S3: Stroke targets predicted using GeneCards and OMIM databases. Table S4: PPI network of intersecting targets. Table S5: GO enrichment terms of intersecting targets. Table S6: KEGG enrichment terms of intersecting targets.
