# Supplementary material for: Volatile Compounds in Musk and Their Anti-Stroke Mechanisms
Source: Metabolites. 2025 Mar 7;15(3):181. doi: 10.3390/metabo15030181 (PMC11943872; doi:10.3390/metabo15030181)
Supplement: Supplementary file 1 [file metabolites-15-00181-s001.zip › metabolites-3487365-Supplymentary Figures.pdf]

Supplementary Figure S1 A: The tuning results of the GC-MS instrument.
Supplementary Figure S1 B: Measurement peak profiles of quality control samples and blank samples.
Supplementary Figure S1 C: Different classifications of VCs.
Supplementary Figure S1 D: Proportion of different-coloured musks in paste musk.
Supplementary Figure S1 E: Construction of the musk-VCs-target network.
Supplementary Figure S1 F: PPI network of intersecting targets.

A

Comprehensive Evaluation: Passed	Evaluation Criteria	Assessment (Actual)
FWHM (69)	Set value within ±0.1u	Pass (0.61u)
FWHM (219)	Set value within ±0.1u	Pass (0.61u)
FWHM (502)	Set value within ±0.1u	Pass (0.60u)
Detector Voltage	Bleow 2.00 kv	Pass (1.77kv)
69/28 Intensity Ratio	Above 2.00	Pass (14.09)
Maximum Mass Drift (69)	Within 0.10u	Pass (0.00u)
Maximum Mass Drift (219)	Within 0.10u	Pass (0.00u)
Maximum Mass Drift (502)	Within 0.10u	Pass (0.00u)
Relative Intensity in High Mass Range (502)	Above 2%	Pass (10%)

B

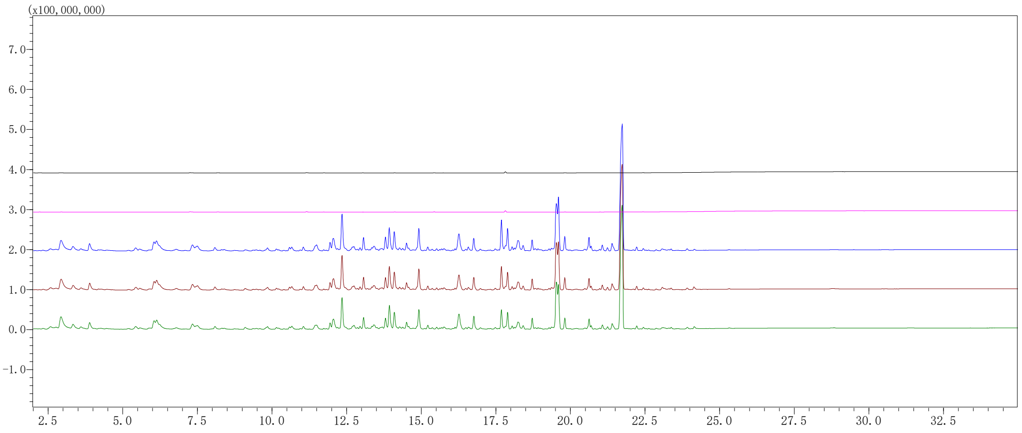

C

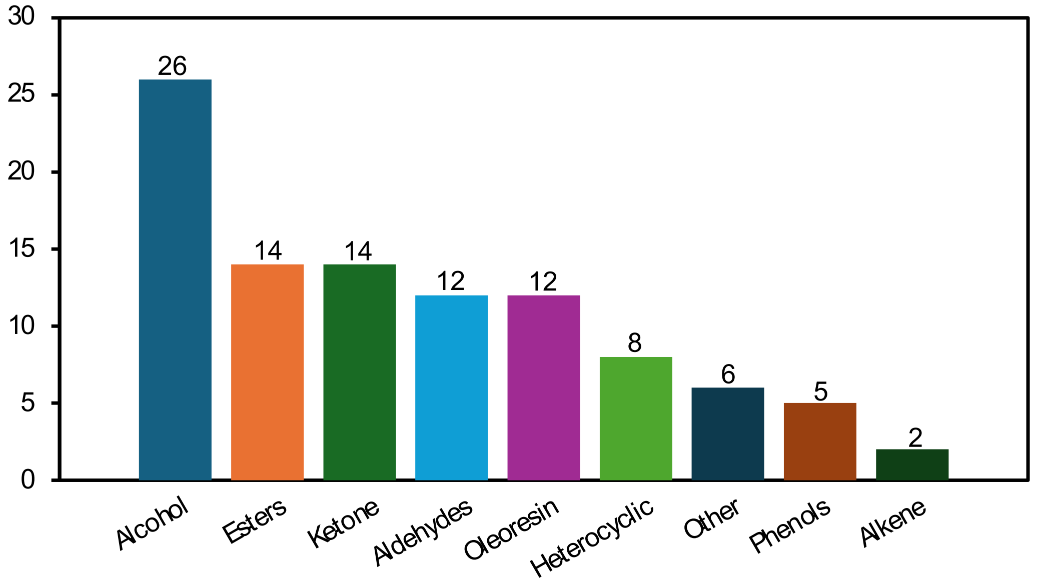
30
25
20
15
10
5
0
26
14
14
12
12
8
6
5
2
Alcohol
Esters
Ketone
Aldehydes
Oleoresin
Heterocyclic
Other
Phenols
Alkene

D

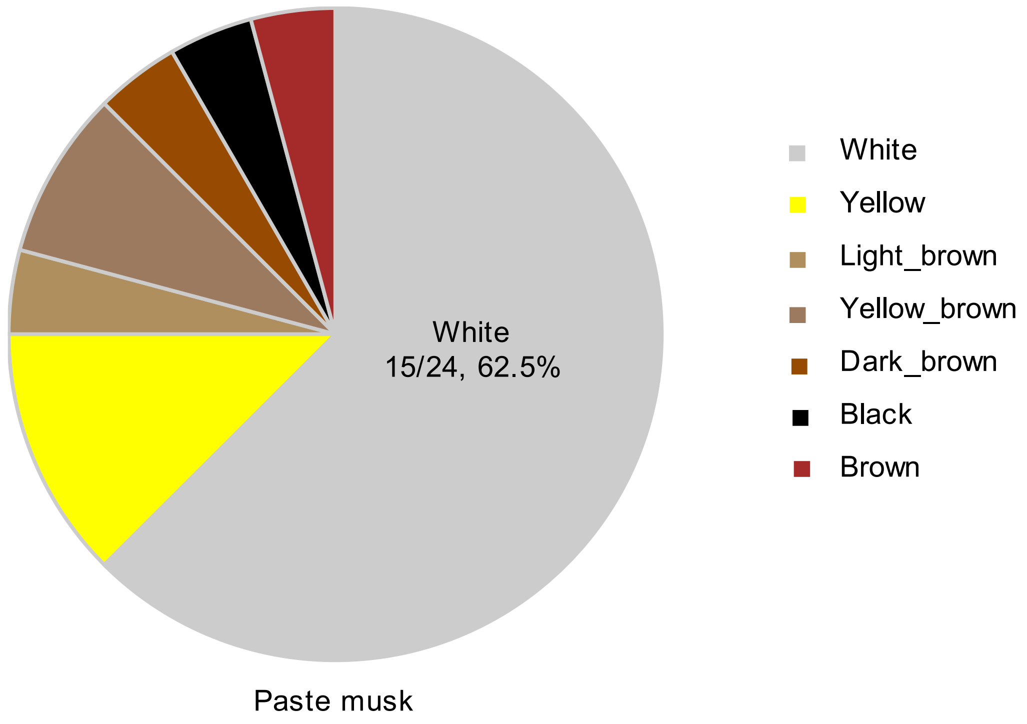
White
15/24, 62.5%
White
Yellow
Light_brown
Yellow_brown
Dark_brown
Black
Brown
Paste musk

E

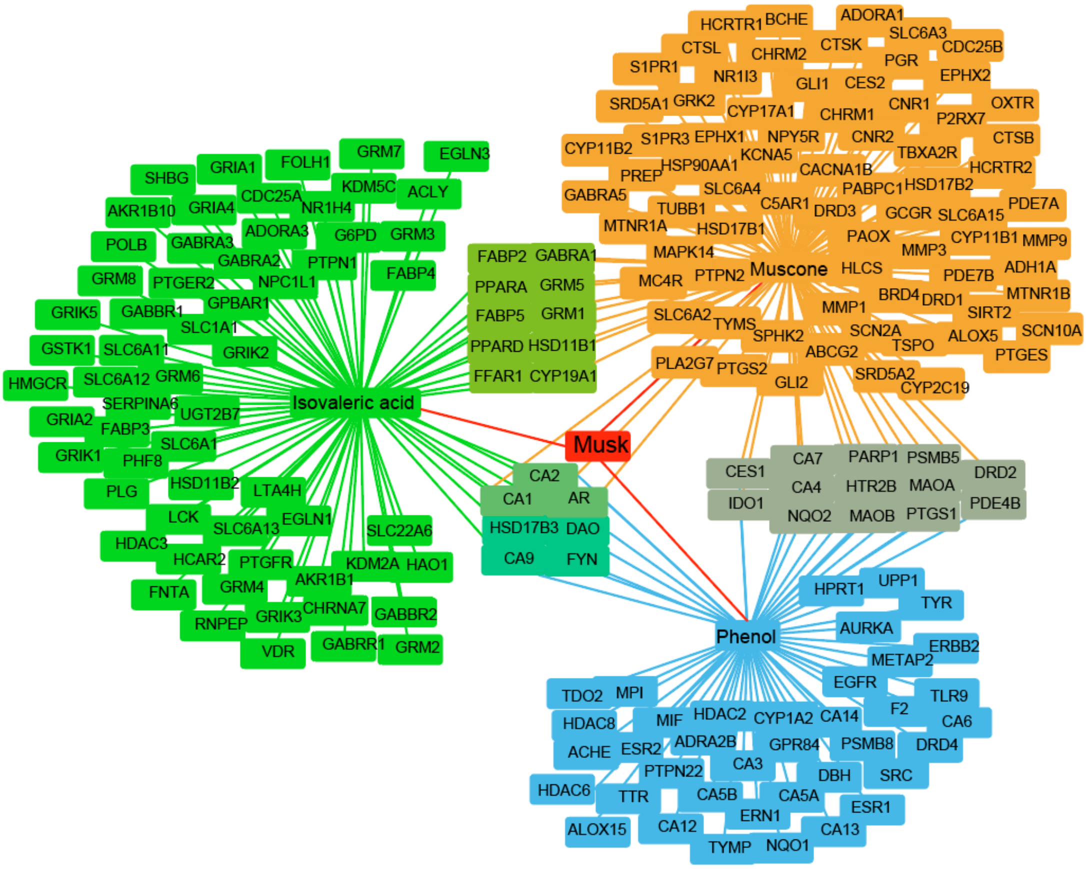

Musk
Isovaleric acid
Muscone
Phenol
SHBG
GRIA1
FOLH1
GRM7
EGLN3
AKR1B10
GRIA4
CDC25A
KDM5C
ACLY
NR1H4
POLB
GABRA3
ADORA3
G6PD
GRM3
GABRA2
PTPN1
FABP4
GRM8
PTGER2
NPC1L1
GPBAR1
GRIK5
GABBR1
SLC1A1
GSTK1
SLC6A11
GRIK2
HMGCR
SLC6A12
GRM6
SERPINA6
UGT2B7
GRIA2
FABP3
SLC6A1
GRIK1
PHF8
HSD11B2
PLG
LTA4H
LCK
SLC6A13
EGLN1
SLC22A6
HDAC3
HCAR2
PTGFR
KDM2A
HAO1
AKR1B1
FNTA
GRM4
CHRNA7
GABBR2
RNPEP
GRIK3
GABRR1
GRM2
VDR
FABP2
GABRA1
PPARA
GRM5
FABP5
GRM1
PPARD
HSD11B1
FFAR1
CYP19A1
CA2
CA1
AR
HSD17B3
DAO
CA9
FYN
HCRTR1
BCHE
ADORA1
SLC6A3
CTSL
CHRM2
CTSK
CDC25B
S1PR1
NR1I3
PGR
EPHX2
GLI1
CES2
SRD5A1
GRK2
CYP17A1
CHRM1
CNR1
OXTR
P2RX7
CYP11B2
S1PR3
EPHX1
NPY5R
CNR2
CTSB
KCNA5
TBXA2R
PREP
HSP90AA1
CACNA1B
HCRTR2
GABRA5
SLC6A4
PABPC1
HSD17B2
C5AR1
PDE7A
TUBB1
DRD3
GCGR
SLC6A15
MTNR1A
HSD17B1
PAOX
CYP11B1
MMP9
MAPK14
MMP3
ADH1A
MC4R
PTPN2
HLCS
PDE7B
BRD4
MTNR1B
DRD1
MMP1
SLC6A2
TYMS
SIRT2
SCN2A
SPHK2
ALOX5
SCN10A
TSPO
ABCG2
PLA2G7
PTGS2
PTGES
SRD5A2
GLI2
CYP2C19
CA7
PARP1
PSMB5
CES1
DRD2
CA4
HTR2B
MAOA
IDO1
PDE4B
NQO2
MAOB
PTGS1
HPRT1
UPP1
TYR
AURKA
ERBB2
METAP2
EGFR
TDO2
MPI
TLR9
HDAC8
MIF
HDAC2
CYP1A2
CA14
F2
CA6
ACHE
ESR2
ADRA2B
GPR84
PSMB8
DRD4
PTPN22
CA3
DBH
SRC
HDAC6
TTR
CA5B
CA5A
ESR1
ALOX15
CA12
ERN1
CA13
TYMP
NQO1

F

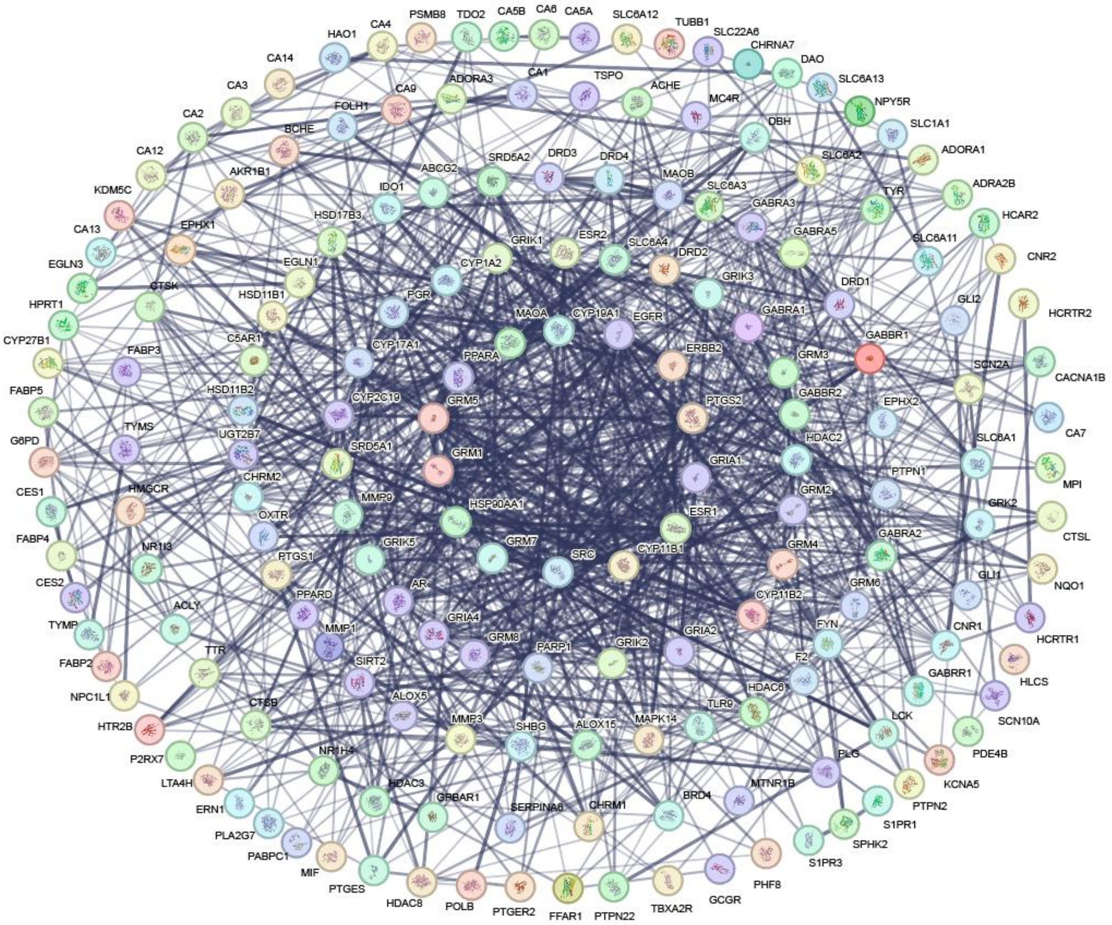
HAO1
CA4
PSMB8
TDO2
CA5B
CA6
CA5A
SLC6A12
TUBB1
SLC22A6
CHRNA7
DAO
SLC6A13
NPY5R
CA14
CA3
CA9
ADORA3
CA1
TSPO
ACHE
MC4R
SLC1A1
CA2
FOLH1
BCHE
DBH
ADORA1
CA12
AKR1B1
SRD5A2
DRD3
DRD4
MAOB
SLC6A2
KDM5C
ABCG2
IDO1
SLC6A3
TYR
ADRA2B
HSD17B3
GABRA3
HCAR2
CA13
EPHX1
GRIK1
ESR2
SLC6A4
GABRA5
SLC6A11
EGLN3
EGLN1
CYP1A2
DRD2
CNR2
CTSK
GRIK3
DRD1
HPRT1
HSD11B1
PGR
GLI2
MAOA
CYP19A1
EGFR
GABRA1
HCRTR2
C5AR1
CYP27B1
CYP17A1
GABBR1
FABP3
ERBB2
GRM3
PPARA
CACNA1B
SCN2A
FABP5
HSD11B2
CYP2C19
GRM5
GABBR2
EPHX2
PTGS2
CA7
TYMS
G6PD
UGT2B7
HDAC2
SLC6A1
SRD5A1
GRM1
GRIA1
MPI
PTPN1
CES1
HMGCR
CHRM2
GRM2
HSP90AA1
ESR1
MMP9
GRK2
FABP4
OXTR
NR1I3
CTSL
GABRA2
GRM7
GRM4
GRIK5
CES2
PTGS1
SRC
CYP11B1
NQO1
GLI1
AR
PPARD
CYP11B2
ACLY
GRM6
TYMP
HCRTR1
MMP1
GRIA4
FYN
CNR1
FABP2
TTR
GRM8
PARP1
GRIK2
GRIA2
F2
HLCS
SIRT2
GABRR1
NPC1L1
SCN10A
ALOX5
HDAC6
CTSB
TLR9
HTR2B
MAPK14
MMP3
SHBG
ALOX15
LCK
PDE4B
P2RX7
NR1H4
PLG
KCNA5
LTA4H
HDAC3
MTNR1B
GPBAR1
PTPN2
ERN1
SERPINA6
CHRM1
BRD4
S1PR1
PLA2G7
SPHK2
PABPC1
S1PR3
MIF
PHF8
PTGES
GCGR
HDAC8
POLB
PTGER2
FFAR1
PTPN22
TBXA2R
